# Comment on Schillberg, E., et al; Distribution of *Ixodes scapularis* in Northwestern Ontario: Results from Active and Passive Surveillance Activities in the Northwestern Health Unit Catchment Area. *Int. J. Environ. Res. Public Health* 2018, *15*, 2225

**DOI:** 10.3390/ijerph16111939

**Published:** 2019-05-31

**Authors:** John D. Scott

**Affiliations:** International Lyme and Associated Diseases Society, 2 Wisconsin Circle, Suite 700, Chevy Chase, MD 20185-7007, USA; jkscott@bserv.com

I am filing three errata pertaining to the article (Schillberg et al., 2018) [1].

**Erratum 1:** Schillberg et al. report an “*Amblyomma cajennense*” tick in central Canada [1]. This foreign tick species lacks specific background information, including (a) history of travel, (b) date tick collected, (c) developmental life stage, (d) tick identifier, (e) identification method (molecular, morphologic), and (f) the depository. With the exception of knowing this tick was collected from a human, no other background information is available on this *Amblyomma* tick. The person who identified this specimen has neither been recognized in the Material and Methods section, nor named in the Author Contributions. The authors overlooked the fact that Nava et al. split the *Amblyomma cajennense* sensu lato complex into six separate tick species [2]. Biogeographically, the closest clade is *Amblyomma mixtum*, which is indigenous from southern Texas to coastal Ecuador. Specifically, *Amblyomma cajennense* sensu stricto (s.s.) is found in the Amazonian region of South America [2]. 

Since *A. cajennense* s.s. is not known to be transported by passerine birds, this tick species must have been imported by a traveller. Since there is no history of travel, this *Amblyomma* sp. is automatically disqualified as a legitimate tick in the Northwestern Health Unit (NWHU) catchment. Moreover, the authors made no attempt to have the tick properly identified by molecular analysis (e.g., DNA barcoding). Five years elapsed from the time the *Amblyomma* tick was collected until it was published; this lengthy time period provided ample time for the authors to have the tick properly identified by molecular analysis. In addition, the authors failed to recognize that Guglielmone et al. listed 57 *Amblyomma* spp. in the Neotropical Region [3], and this specimen could easily have been another one of these *Amblyomma* species. Not only did the authors overlook other tick species, they misguidedly listed “*Amblyomma cajennense*” in Table 1. As it stands now, the identification of this ambiguous *Amblyomma* tick is an unsolved mystery and, because the tick is no longer available for identification, it will forevermore remain an obscurity. Because of the substandard identification procedures, “*Amblyomma cajennense*” is an invalid entry in Table 1.

**Erratum 2:** Schillberg et al. show that Kenora is a Lyme disease hotspot, but ignored related tick−host−pathogen information documented by Scott et al. [4]. This particular enzootic study revealed nine tick species in close proximity to Kenora, Ontario, and eight of these ixodid ticks were infected with the Lyme disease bacterium, *Borrelia burgdorferi* sensu lato (Bbsl). Of these eight Bbsl-infected tick species, five species, namely *Ixodes angustus*, *Ixodes banksi* (beaver tick), *Ixodes cookei* (groundhog tick*), Ixodes muris* (mouse tick), and *Ixodes scapularis* (blacklegged tick; northern populations previously considered *Ixodes dammini*) [5], are human-biting ticks. Significantly, Scott et al. showed that the mustelid tick, *Ixodes gregsoni*, has shifted its home range westward by 200 km juxtaposed to Kenora, Ontario and, as a Lyme-carrying tick, is interconnected enzootically with wildlife in the Kenora District [4]. Failure to cite Scott et al. [4], which highlights eight species of ticks that harbour Bbsl in the Kenora area, is a misrepresentation of ecological and epidemiological facts. Moreover, this article was sent to the Medical Officer of Health (K.N.Y.H) shortly after it was published. Although this peer-reviewed scientific article was acknowledged by the NWHU office staff shortly after publication, Scott et al. was not cited. 

Schillberg et al. report an established population of *I. scapularis* in the vicinity of Kenora, Ontario, and presumably reported a blended Bbsl infection prevalence of 59.8%. The authors neither provide any specific collection sites, nor do they provide any individual Bbsl prevalence by location. In comparison, Scott et al. documented a mean Bbsl infection prevalence of 73% (blacklegged tick adults) at a fixed site on Corkscrew Island, which is located 20 km southwest of Kenora, Ontario. Notably, Bbsl infection prevalence, by season, was also provided in Scott et al. [4]. 

Although Schillberg et al. mentioned that more studies are needed on forest composition, other researchers have already addressed this arboreal point. In fact, Scott et al. specifically addressed the ecological value of bur oak as it pertains to ticks, small mammals, and white-tailed deer, *Odocoileus virginianus* [4]. An abundant crop of acorns provides energy-rich food for mice and deer. It is noteworthy that a grove of bur oaks acts as suitable habitat for the enzootic transmission cycle of Bbsl and, importantly, supports and perpetuates all life stages of *I. scapularis* ticks and their hosts.

In addition, Scott et al. provided pathological and neurological manifestations of Lyme disease [4], and made available helpful information for patients and health care providers practicing in this area. By not citing Scott et al., Schillberg et al., who are all public health officials, ignored this vital clinical information. By law, it is mandatory that senior public health unit staff inform the public about any infectious disease area, such as a Lyme disease endemic area. Public notification of such sites and posting warning signs are legally required.

Also, Schillberg et al. only report five valid tick species in the NWHU catchment [1]. In contrast, Scott et al. reported nine indigenous tick species in the Kenora District, and eight of these tick species were positive for Bbsl [4]. From a public health standpoint, each of the five Bbsl-infected tick species have vector competence and bite humans [4]. The omission of Scott et al. is not only a glaring oversight [4], it is an ostensive misrepresentation of the facts.

**Erratum 3**: Schillberg et al. cited several tick and climate change references that misrepresent when *I. scapularis* populations were studied and when they were established [6,7,8,9,10,11]. The baseline climate change maps report no *I. scapularis* ticks in northwestern Ontario and southeastern Manitoba before 2000, and yet, just across the Ontario–Manitoba border at Marchand, Manitoba, Galloway et al. reported blacklegged ticks (two larvae, one nymph), in 1991, parasitizing small mammals at one location [12]. Collectively, these life stages signify an established population [13]. Furthermore, the only way that these larvae could have been there is if a gravid female laid eggs that hatched to unfed larvae. Of note, larvae roam a maximum of 2 m from the egg laying site [14]. Despite this ecological discovery, the tick and climate change researchers took a dismissive approach to these important published findings [6,7,8,9,10,11], and proceeded to design computer models that were hypothetical. Moreover, this trivializing philosophy countered the high prevalence of Bbsl antibodies in the human population in central Canada [12]. As a result, the computer model exhibited the absence of any established population in the area, and showed a gradual tick expansion northward with climate change. This particular approach would explain to the public a reason for not tackling this serious health care issue earlier. The tick problem was programmed for the future. Thus, ill-founded statistical analyses culminated in fabricated erroneous data and, ultimately, resulted in a series of maps that turned out to be non-conclusive. 

Schillberg et al. state that the first known population of *I. scapularis* was at Long Point, Ontario. With more than 70 established populations of *I. scapularis* across Ontario, it is happenstance that Long Point was the first one studied. Based on the Bbsl infection prevalences (Corkscrew Island, 73%; Long Point, 67%), there was a miscalculation in the computer model that resulted in a geographical, north-south inversion. Since tick abundance and the infection prevalence of Bbsl increases with time in an incremental, see-saw pattern [15,16], the establishment of *I. scapularis* on Corkscrew Island pre-dates Long Point. By programming the computer models on when the sites were studied rather than when they were established, the tick and climate change authors produced incorrect maps. The absence of *I. scapularis* on the climate change maps prior to 2000 in the NWHU catchment area are not only deceptive [6,7,8,9,10,11], they are free range guesstimates and obfuscations. 

Despite any effect of climate change, it is noteworthy that northward-migrating songbirds transport larval and nymphal blacklegged ticks annually into Canada during spring movement. Also, passerine migrants transport *I. scapularis* southward during fall migration. In these bi-directional migrations, a significant percentage of these ticks are infected with Bbsl. Counterpoint to Schillberg et al. [1], tick dispersal by songbirds during spring migration is directly attributed to phenology—not climate change. In the seven cited references on ticks and climate change [6,7,8,9,10,11,17], the associated authors for all these articles were unable to substantiate an interconnecting link between tick numbers and climate change. Schillberg et al. state that favourable changes in climate will allow more ticks to survive and establish tick populations. However, these authors display considerable uncertainty about whether the climate change models actually support any possible *I. scapularis* expansion.

Schillberg et al. predict that the average temperature in northwestern Ontario will gradually increase by 7 °C in the next 61 years (viz., 2080) [1]. In contrast, the historical mean daily temperature in central Canada has only increased by +0.5 °C in the past 80 years. It appears that the authors’ hypotheses on temperature increase is based on the United Nations International Panel on Climate Change computer forecasts that have been consistently wrong [18]. Contrary to references cited by Schillberg et al., warmer winters do not equate to increased tick numbers. In fact, Scott and Scott have revealed that when daytime temperatures completely melt snow cover in late winter in wooded areas, followed by a sudden drop in overnight temperatures to −12 °C or lower, the overwinter survival of *I. scapularis* adults was significantly reduced [19].

Certain tick and climate change researchers, which were cited by Schillberg et al., state that it was too cold for blacklegged ticks to become established in the Kenora area [7,17], and yet, 100% of the *I. scapularis* larvae in the Lindsay et al. study overwintered successfully [17]. Not only did this tick survival study contain diametrically opposing statements, it started a cascade of misinformation about the ability of *I. scapularis* to overwinter successfully in central Canada. Based on this paradoxical study, public health officials and health care practitioners told the public that it was too cold for *I. scapularis* to survive and establish in northern Canadian environments and, thus, patients could not have Lyme disease. This ill-founded advice had dire consequences for patients. Either patients with tick-borne diseases had to leave the province for diagnosis and treatment, or they had to suffer unending morbidity from these zoonoses. Patients experienced devastating and tragic consequences due to loss of income, loss of schooling, loss of employment, loss of relationships, and loss of property and possessions. Most patients have had to endure profound fatigue, crippling pain, and cognitive impairment. Others have prematurely taken their lives or have had fatal outcomes. In order that patients are diagnosed and treated in a timely manner, the regulating medical colleges must put forward regulations to protect physicians and nurse practitioners, and allow trained health care professionals to treat patients with tick-borne zoonotic diseases efficaciously. These clinicians should have at least 30 continuing medical education (CME)-based credits administered by the International Lyme disease and Associated Diseases Society (ILADS) or have equivalent training in diagnosing and treating tick-borne diseases.

Schillberg et al. purport that climate change is a driver in tick numbers and tick expansion. They also state that there was a general increasing trend in *I. scapularis* submissions each year, and suggest that climate change may be a factor. Nowhere in the ’Data Variables and Sources’ section and the subsequent ’Results’ section is there any data to substantiate a direct relationship between *I. scapularis* ticks and climate change. Furthermore, the references and sub-references do not provide any substantive evidence that climate change increases tick numbers and tick expansion. In reality, public awareness turns out to be the key factor in determining tick numbers; the more ticks people submit for identification and testing, the higher the numbers will be. Any government-funded research on ticks and climate change has been inconclusive.

Schillberg et al. briefly mention that migratory birds may be a factor in tick expansion. However, these authors only mention birds in one sentence, and cite an in-house author; failure to cite other North American researchers is myopic and a misrepresentation of facts. When it comes to tick expansion continent-wide, migratory passerines are the most important factor in the wide dispersal of bird-transported ticks [20]. East of the Rocky Mountains, songbirds transport bird-feeding ticks, including *Amblyomma americanum* (lone star tick), *Haemaphysalis leporispalustris* (rabbit tick), *Ixodes dentatus* (rabbit-associated tick), *Ixodes muris* (mouse tick), and *I. scapularis* (blacklegged tick). Notably, all of these bird-feeding ticks are known to harbour tick-borne pathogens [20]. No computer model could ever be programmed to show when and where a heavily infested songbird would drop bird-feeding ticks and initiate a new established population. Bird movements work independently to climate change. When it comes to ticks, climate change is a non-issue. 

Schillberg et al. report that there is no correlation between forest composition and *I. scapularis* populations [1], and yet, they believe that more studies are needed. In reality, any such exercise would be redundant. Although forest composition is an environmental factor, changing it would be impractical in the NWHU catchment—a massive, Precambrian wilderness area. 

Regardless of where one lives on this planet, seasonal variation is a fact of life. However, Schillberg et al. confuse seasonal variation with climate change in the NWHU catchment. For example, bur oak will initiate flower buds during hot, dry weather in August. The following year, bur oak will increase mast production and, subsequently, mice and deer will have an abundance of acorns as energy-rich food. As a result, more mice and deer will support the reproduction of larval, nymphal, and adult *I. scapularis* ticks. Increased tick numbers will exacerbate the enzootic transmission cycle of Bbsl, and increase the public health risk. Also, despite any global warming, photoperiod is a limiting abiotic factor for *I. scapularis* establishment, especially in regions north of 52° latitude [21].

Schillberg et al. report an average of two human Lyme disease cases (2011−2017) per year in the NWHU catchment, which suggests that under-detection and under-reporting by the medical profession in this area is a problem. It is hard to imagine that this rearward-looking approach is the mandate of public health. 

Once established in the human body, Lyme disease can be an insidious, persistent disease. Therefore, it is paramount to treat this zoonosis early. Bbsl slips by the immune system, and sequesters in deep-seated tissues. If inadequately treated, this spirochetosis becomes refractory and, ultimately, can result in fatal outcomes [22]. Chronic Lyme disease is a fact of life [23]. Most importantly, medical professionals need to update their differential diagnosis on tick-borne zoonoses. 

## Conclusions

In all, 53 troubling points were noted throughout the article, including invalid entry of tick data, omission of key ecological and epidemiological information, and misrepresentation of facts about ticks and climate change. The authors state that they want to mitigate the Lyme disease problem in the NWHU catchment area, but they offer no viable solution for this pernicious, zoonotic disease. Because the vast majority of health care practitioners are not recognizing Lyme disease and associated tick-borne zoonoses, the existing health care situation is not only untenable, it is outside the code of professional and medical ethics. There is a practical medical solution: Lyme-literate health care professionals are needed throughout the province of Ontario who have at least 30 ILADS-based CME credits in Lyme disease and associated tick-borne diseases, and can diagnose and treat suspect human cases efficaciously, as clinically required.
